# A Statistical Model of COVID-19 Infection Incidence in the Southern Indian State of Tamil Nadu

**DOI:** 10.3390/ijerph191711137

**Published:** 2022-09-05

**Authors:** Tanmay Devi, Kaushik Gopalan

**Affiliations:** Department of Computing and Data Sciences, FLAME University, Pune 412115, India

**Keywords:** COVID-19 infection incidence, machine learning, statistical modeling of COVID-19

## Abstract

In this manuscript, we present an analysis of COVID-19 infection incidence in the Indian state of Tamil Nadu. We used seroprevalence survey data along with COVID-19 fatality reports from a six-month period (1 June 2020 to 30 November 2020) to estimate age- and sex-specific COVID-19 infection fatality rates (IFR) for Tamil Nadu. We used these IFRs to estimate new infections occurring daily using the daily COVID-19 fatality reports published by the Government of Tamil Nadu. We found that these infection incidence estimates for the second COVID wave in Tamil Nadu were broadly consistent with the infection estimates from seroprevalence surveys. Further, we propose a composite statistical model that pairs a k-nearest neighbours model with a power-law characterisation for “out-of-range” extrapolation to estimate the COVID-19 infection incidence based on observed cases and test positivity ratio. We found that this model matched closely with the IFR-based infection incidence estimates for the first two COVID-19 waves for both Tamil Nadu as well as the neighbouring state of Karnataka. Finally, we used this statistical model to estimate the infection incidence during the recent “Omicron wave” in Tamil Nadu and Karnataka.

## 1. Introduction

India, like the rest of the world, has suffered severe consequences from the COVID-19 pandemic from a public health as well as economic perspective. As with many infectious diseases, the level of infection is commonly underestimated since a significant number of individuals infected remain undetected, either because they are asymptomatic or have very mild symptoms, and hence do not get tested and reported [1]. There may also be disadvantaged or neglected groups of the population who are less likely to receive healthcare or testing. Case underdetection may be aggravated during an epidemic because testing capacity may be limited and restricted to those with severe illnesses and priority risk groups that include front-line healthcare workers, elderly people and individuals with comorbidities [2].

Without a reliable assessment of infection incidence, it is difficult to forecast the true impact of COVID-19 in any particular vulnerable population, which may hinder policy development and have major ramifications for decision-making in the future. Access to credible estimates of the true number of infections is critical for risk assessments and developing effective solutions. Due to the unavailability of timely access to representative samples, reliable approaches for estimating the number of infections during an epidemic are required, particularly for transferring internationally developing knowledge to local contexts where such data are lacking [3]. As a result, quantifying the infection incidence is crucial for every region in order to analyse COVID-19 health inequalities in the community and influence public health, health system, and community-based interventions. This study attempts to estimate infection incidences for the Indian state of Tamil Nadu across waves of COVID-19.

There have been many attempts to infer this true infection incidence of COVID-19 from deaths, confirmed cases, tests, and sero-surveys. Previous studies have used observable data sources such as cases, fatalities, tests, and sero-surveys to create a basic Bayesian framework for estimating infection incidence [4,5]. Moreover, susceptible-exposed-infectious-recovered (SEIR)-based model has been used as well to estimate the infection incidence [6]. Similarly, infection incidences is also widely estimated using the infection fatality ratio (IFR) based on current seroprevalence data to infer the number of true infections and age-wise fatalities. IFR refers to the ratio of COVID-19 fatalities relative to the number of true COVID-19 infections within a population. While the case fatality ratio (CFR) is a more generally used metric and is well understood by specialists, IFR provides key information for policymakers, especially given the huge fluctuation in CFR estimates. However, since the number of true infections is not directly observable, IFR must also be inferred indirectly through statistical methods. Further, the importance of age-specific IFRs has been emphasised in determining public policy in a systematic survey of 27 studies conducted across the globe [7]. In the Indian context, publicly accessible data on reported number of cases and national seroprevalence surveys have been utilised to estimate an under-reporting factor of 11.11 and 26.77 for Wave-I and Wave-II infections, respectively [8]. However, the above study did not include age-split or sex-wise distribution based on the seroprevalence rate and fatalities to estimate true infections. To address this, age-specific IFR using data from seroprevalence surveys in Mumbai and Karnataka as well as a random sample of economically disadvantaged migrants in Bihar were estimated [9]. In general, detailed age/sex-specific fatality data are not accessible in India. However, Tamil Nadu is one of the few states with daily media COVID-19 bulletins available in conjunction with age-specific sero-surveys, allowing us to estimate IFR-based infection incidences.

Therefore, through our study, using seroprevalence data, age/sex wise fatality data, and cases, we estimate the infection fatality ratio and infection incidences for the state of Tamil Nadu for Wave-I and Wave-II.

Although a seroprevalence survey assists in calculating IFR for earlier waves of the virus, the same methodology is not feasible for subsequent waves, on account of vaccinations leading to a change in the relationship between infections and fatalities. After large-scale vaccinations and the changing severity of variants, the correlation between infection and fatalities is significantly weakened. The Union Health Ministry of India reported that one vaccination dose alone is 96.6% effective in avoiding COVID-19 fatalities, while two doses are 97.5% effective, after analysing fatality data for Wave-II [10]. Since fatality data are the foundation of IFR computation, newer techniques for infection incidence calculation must be explored. Chiu and Ndeffo-Mbah [11] hypothesised that passive case detection resulted in preferential diagnostic testing for those at greater risk of infection, and that this could be represented as a convex function of the overall testing rate. They illustrated that as the incidence of test positive rate (TPR) rose in the community, so did the prevalence of undiagnosed infections. Thus, we developed a statistical model to estimate infection incidences for Wave-II and Wave-III (Omicron wave) in Tamil Nadu based on observed data (cases, test positivity ratio (TPR)) excluding fatalities.

## 2. Materials and Methods

Estimating infection fatality rates necessitates a reliable assessment of the number of infections and fatalities caused by COVID-19 in the community. As a result, the age- and sex-wise split of fatality data in this study were obtained by utilising the Health and Family Welfare Department of the Government of Tamil Nadu’s (TN) daily media bulletins [12]. The media bulletins are delivered in PDF format (with death cases including age, sex, and date), and the text information was extracted using the Python programming language’s pdfminer package. For each “Death Case” in the media bulletin, we collected the case number, date of death, and age of the deceased using relevant keyword searches. The data for this study were extracted for the period of 16 May 2020 to 30 June 2021. Out of the 410 days in this time frame, we were able to process 407 daily bulletins; the rest were either unavailable on the website or were formatted such that our software was unable to process them.

From the 19th of October to the 30th of November 2020, the Tamil Nadu state government conducted population-level seroprevalence surveys in all districts of the state (excluding Chennai). Selvavinayagam et al. [13] presented estimates of seroprevalence for Tamil Nadu by district, demographic category, and urban status. We used the seroprevalence survey disaggregated by sex and age in this study. The Tamil Nadu seroprevalence rates were separated by age (10-year age bins) and sex.

Finally, to develop our statistical model, we utilised data from the crowdsourcing website covid19india.org for COVID-19 reported cases and test positivity ratio (TPR) in Tamil Nadu (TN) and Karnataka (KA).

### 2.1. IFR Calculation

First, we computed the age- and sex-based fatalities over Wave-I (1 June 2020 to 30 November 2020) using the daily bulletins published by the Government of Tamil Nadu. The cumulative deaths for the respective age (10-year age bins as in the sero-survey) and sex during the previous 6 months (1 June 2020 to 30 November 2020) were considered. Shioda et al. [14] showed that within 6 months after seroconversion, more than 85% of infected people were projected to be seronegative due to declining antibodies. Immunoglobulins targeting severe acute respiratory syndrome coronavirus 2 (SARS-CoV-2) have been observed to wane below the detectable level of serologic tests, hence the time period is believed to be just 6 months. In addition, infection incidence was calculated based on Census of India 2011 [15] age-wise (%) population distribution estimates according to the age-wise split in the sero-survey. For example, individuals aged 18–29, constituted 20% of TN’s population, with a seroprevalence of 30.4% after Wave-I. As a result, the total infection incidence in this age group was 30.4% of 20% of the entire population of Tamil Nadu. The infection incidences based on seroprevalence rate were divided by the cumulative fatality figures divided in identical 10-year bins. For all x∈D where *D* is the set of all age- and sex-disaggregated demographic groups,

(1) $$IFR\left(x\right)=\frac{\sum_{t\in Wave1}f\left(x,t\right)}{I_{sero}\left(x\right)}$$

where IFR(x) is the estimated infection fatality ratio for demographic group *x*, f(x,t) is the number of fatalities from demographic group *x* on date *t*, and $I_{sero}\left(x\right)$ is the number of infections estimated by the sero-survey for demographic group *x* for Wave-I. The variable *t* was iterated over all dates in Wave-I. This resulted in age-based and sex-specific IFR for each age group, as seen in Table 1.

Furthermore, in order to get IFR-based daily estimates of infection incidence, daily fatalities (15-day lag) split in the above age categories were divided by the aforementioned *IFR* rates as follows.

(2) $$I_{est}\left(t\right)=\sum_{x\in D}\frac{f(x,t)}{IFR(x)}$$

where *D* is the set of all demographic groups, $I_{est}\left(t\right)$ is the number of estimated infections on date *t*, and f(x,t) and IFR(x) retain the same definitions as Equation (1). It is important to note that we estimated the number of new infections occurring every day, not the number of active cases. In this regard, we assumed a fixed lag of 15 days between onset of infection and fatality. This is implausible and untested but should not introduce significant biases in the larger statistics. We demonstrate evidence of the plausibility of these estimates in the “Results” section.

### 2.2. Statistical Model for Infection Incidence

With the advent of widespread COVID-19 vaccination among the population, the relationship between daily fatalities and the underlying infection incidence has become weaker and thus more complex to estimate. Further, with improvements in COVID-19 treatment protocols over time, it is no longer defensible to try to estimate infection incidence by applying the IFRs determined in the first wave on recent fatality data. However, the estimation of infection incidence still plays a crucial part in making public policy decisions such as school closures and openings, mask mandates, and other public interventions meant to control the spread of COVID-19. Thus, it is important to find alternative techniques to estimate infection incidence without relying on the assumption of a strong relationship between COVID-19 fatalities and infections. In this section, we propose an empirical model using daily reported cases and the TPR to provide crude estimates of daily infection incidence in the population.

The relationship between daily reported cases and corresponding new infections occurring on that date can be represented by the equation:

(3) I(t)=μ(t)·c(t)

where I(t) corresponds to the new infections occurring on a given day, c(t) is the number of COVID-19 cases detected on that day, and μ(t) is the ratio between the two. In practice, there is a variable lag between acquiring infection and testing positive for COVID-19; but we ignored this lag for the purpose of this analysis. We emphasise here that there was reason to expect that the ratio between infections and detected cases was highly variable over the course of the pandemic. We expected that a higher fraction of infections would be detected while the pandemic was under control, whereas we expected a higher fraction of undetected cases during periods of peak surge when the medical and public health systems were under significant strain. One indicator of the instantaneous ratio between infections and cases is the daily test positivity ratio (TPR), which is the ratio of tests conducted on any given day that are reported positive. A high value of TPR (which occurred during COVID-19 peak surges) suggests that only a small fraction of infections is being detected at that point in time, whereas a low value of TPR is indicative of a more effective surveillance of infections. The prevalence of undetected infections in the population rises as the test positivity rate rises in the population [11].

We used a k-nearest neighbours regression model [16] to estimate μ(t) using TPR and daily reported cases as the predictor inputs. We used one-third of the available data—selected randomly—as the training set. The training set consisted of data from 133 days with daily TPR and daily reported cases as inputs and μ(t) as the predicted value. We used Euclidean distances to compute the 2 nearest neighbours from the training set for each observation and assigned the average of their μ values as the estimated μ for the observation. The averaging assigned uniform weights for each neighbour regardless of the distance to the observation being considered.

One characteristic of the k-nearest neighbours model is that it is not valid for cases where the predictor variables lie outside the range of predictors seen in the training set. To address this issue, we paired the k-nearest neighbours model with a power-law characterisation of the relationship between *TPR*, reported daily cases, and μ(t) for those data paints which lie outside the range seen in the training set. We found through observation of TN incidence and case data that the ratio μ(t) could be characterised empirically as follows:

(4) $$\mu \left(t\right)=15.6^{1+TPR(t)}$$

where TPR(t) is the daily test positivity ratio for the given day. It has a maximum value of 1 (when all tests conducted for the day are reported positive) and a minimum value of 0 (when all tests are reported negative). Thus, we propose a way of crudely estimating infection incidence using easily observable and publicly reported variables. To substantiate our statistical model, we used the infection fatality ratio (IFR) by age and sex for the neighbouring state of Karnataka determined by Cai et al. [9,17], in addition to those we derived for Tamil Nadu. A similar process to obtain daily infection incidences followed for TN was employed for Karnataka. The results from these analyses are described in the next section.

## 3. Results

In the previous section, we described the procedure used to derive age- and sex-wise IFR for the state of Tamil Nadu. These estimates are presented in Table 1. Overall IFR for the state of Tamil Nadu was 0.078% for Wave-I, which was comparable to the estimate by Levin et al. (2022) for Karnataka (0.08%) but lower than their estimate for Chennai [18]. We must note here that undetected COVID-19 fatalities would mean that our estimates of IFR were likely to be underestimated; however, the effect of this would cancel out in Equation (2) as both the numerator and denominator would be similarly underestimated. Thus, our estimates of infection incidence were relatively insensitive to potential undercounting of COVID-19 fatalities, even if the IFR values reported in Table 1 were sensitive to undercounting errors.

**Figure 1:**
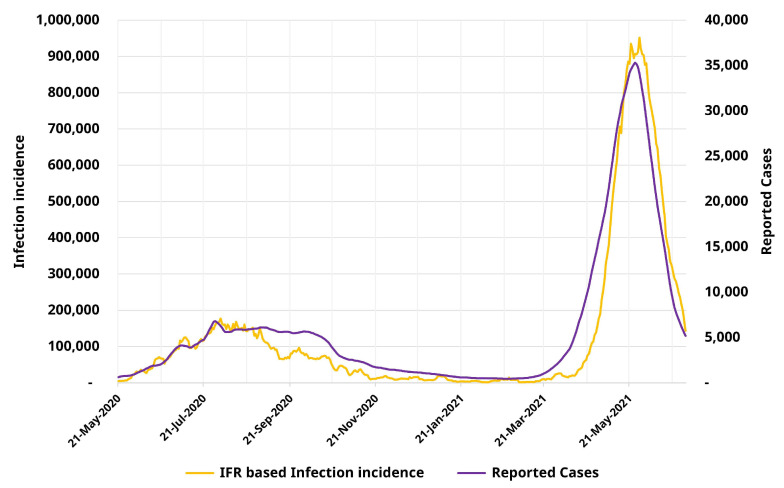
Time-series graph of reported cases and IFR-based infection occurrence estimates for Tamil Nadu from 21-May-2020 to 30-June-2021.

We previously described the methods used to derive daily estimates of new COVID-19 infections for Tamil Nadu. First, we demonstrated that our estimates for daily infections followed a similar temporal pattern to that of the reported cases. Figure 1 depicts the time series of reported cases and infection occurrences—revealing broadly similar trends in both variables, with cases accounting for approximately 1 in 15 infections. Further, we observe that using IFR estimates derived solely from Wave-I, estimates for Wave-II also follow a similar trajectory to that of the reported cases.

Additionally, seroprevalence estimates for Tamil Nadu post-Wave-II (1st March 2021 to 30th June 2021) were calculated from 28 June to 7 July 2021 with an overall seroprevalence rate of 67.10% [13]. In Table 2, we compare the infection incidence estimates for Wave-II derived using the IFR calculations with the corresponding estimates from the sero-survey for each age category. The differences are highest (51%) for the 30–39 age group and the lowest for the 50–59 age group (7%). Overall, the IFR-based estimates are higher than the sero-survey estimates by ≈14%. These differences could arise due to several factors. Most significantly, any significant undercounting of fatalities in the official estimates would lead to artificially low estimates of the IFR and consequently an overestimation of the number of infections for a given number of fatalities.

**Figure 2:**
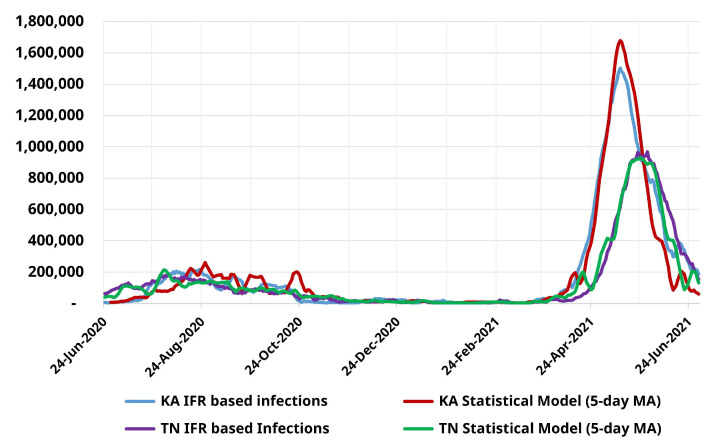
Comparison between the statistical model and IFR-based infection occurrence estimates for Tamil Nadu and Karnataka from 24-June-2020 to 30-June-2021.

Figure 2 plots the time series of IFR-based infection incidence estimates from Tamil Nadu and Karnataka for both the first and second waves along with the corresponding estimates from the TPR-based statistical model. We find that the statistical model matches the IFR-based estimates to a high degree. In Tamil Nadu, the peak number of reported infections are estimated to be approximately 210,000 (930,000) in Wave-I (Wave-II) which corresponds to an overestimation (underestimation) of 15% (4%) relative to the IFR based estimates. Similarly, the highest number of estimated infections in Karnataka are ≈260,000 (1.7 million) for Wave-I (Wave-II) compared to ≈217,000 (1.5 million) from IFR-based estimates; an overestimation of 20% (11.7%). We note that while the match between the statistical model and IFR-based estimates is variable and inexact, the total number of infections estimated by the statistical model lies within ≈3% of those from the IFR-based estimates for both Tamil Nadu and Karnataka.

**Figure 3:**
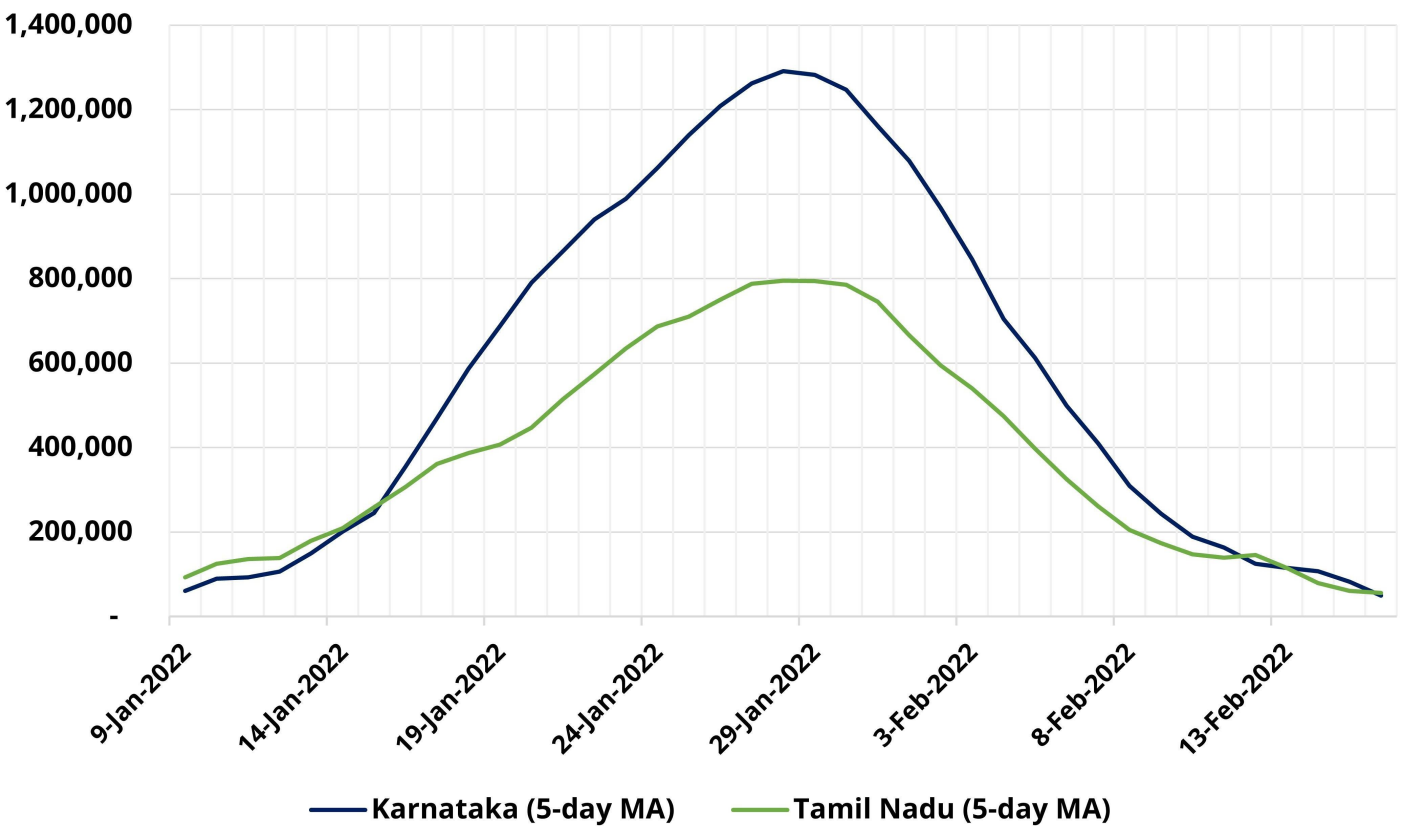
Statistical-model-based infection occurrence estimates for Tamil Nadu and Karnataka for Wave-III from 5-January-2022 to 15-February-2022.

Figure 3 shows the estimated infections in Tamil Nadu and Karnataka for the “Omicron Wave” in India, based on our statistical model for 5-January-2022 to 15-February-2022. The cumulative estimated infections for Tamil Nadu are ≈15.4 million cases for this period with the peak number of estimated daily cases at ≈790,000. Our model suggests that reported cases account for only 1 in 23 infections in this wave, broadly similar to the first two waves. On the other hand, our model suggests that Karnataka had over 23 million infections during the corresponding period, approximately 25 times the number of reported cases. These estimates were not validated due to the lack of available validation data sources and hence must be used with caution. However, they are consistent with the global experience of rapid spread of COVID-19 due to the Omicron variant.

## 4. Discussion

This manuscript contains a detailed analysis of COVID-19 infection incidence in the Indian state of Tamil Nadu. We used seroprevalence survey data along with cumulative COVID-19 fatality reports from the first COVID-19 wave in Tamil Nadu—1 June 2020 to 30 November 2020—to estimate age- and sex-specific COVID-19 infection fatality rates for Tamil Nadu. This methodology has been used extensively globally and our IFR estimates are broadly consistent with those estimated in other parts of the world. Next, we used the daily COVID-19 fatality rates in conjunction with the estimated IFRs to estimate the rate of new daily infections in Tamil Nadu for the first and second COVID waves. We found that these estimates for Wave-II were broadly consistent with the infection estimates from seroprevalence surveys; we emphasise here that Wave-II seroprevalence data were not used for our IFR estimates and hence could be considered an independent validation source. However, we did not use fatality data to estimate infections post-June 2021 since widespread COVID-19 vaccination in the population had resulted in greatly weakening the link between COVID-19 infection and fatalities. To address this constraint, we proposed a composite statistical model to estimate COVID-19 infection incidence based on observed cases and test positivity ratio using a k-nearest neighbours model to estimate daily infections when the observations fell within the range of those found in the training dataset. For observations that fell outside the range of the training dataset, we utilised a power-law characterisation to estimate the infection incidence. We found that that model matched closely with the IFR-based infection incidence estimates for both Wave-I and Wave-II for both Tamil Nadu as well as the neighbouring state of Karnataka. Finally, we used this statistical model to estimate infection incidence during Wave-III of the pandemic—the “Omicron wave”—in Tamil Nadu and Karnataka.

The composite model proposed in this manuscript has several advantages. k-nearest neighbour regression offers a computationally efficient means of transforming higher dimension data into a one-dimension space and is agnostic to the dimensionality of the predictor dataset [17]. It does not require explicit parametrisation of the relationship between the predictor and predicted variables as long as the predictor variables lie with the ranges observed in the training dataset. To account for “out-of-sample” data points, i.e., data points that lie outside the ranges observed in the training dataset, we used a power-law relationship to characterise the infection incidence. We found that this extrapolation model reasonably characterised the infection incidence in the second COVID-19 wave, where the peak values substantially exceeded all previously observed values. Thus, we found that the proposed model provided a robust and computationally efficient means to estimate the infection incidence at all stages of the pandemic, which was the main objective of the study.

A few limitations of our analysis must be noted here. First, our analysis assumed that the seroprevalence surveys that we used provided accurate estimates of the infection incidence among the population and that they did not contain biases between different age groups and sexes. Second, we did not account for differing severities of different variants of COVID-19 in our analysis. The impact of this variability was seen statistically in our IFR-based estimates of infection incidence in the second COVID-19 wave (“Delta” wave) where our estimates of infection incidence significantly exceeded those derived from the sero-survey for the 30–39 and 40–49 age groups. This error could be attributed in large part to higher mortality rates during the Delta wave compared to the previous wave [19] (the difference in mortality was more significant in middle-aged patients compared to the elderly); thus, a given number of fatalities would be caused by significantly fewer infections during the Delta wave compared to the earlier COVID-19 wave. Since our study calculated IFRs from the data from the first wave, we significantly underestimated the number of fatalities per infection caused by the Delta variant and conversely overestimated the infections corresponding to the fatalities observed during the second COVID-19 wave.

Additionally, our analysis did not address the heterogeneity in infection incidence between different districts, which could be quite significant. Finally, the accuracy and validity of our statistical model was validated only indirectly using IFR-based infection incidence estimates. Further data and validation are required to adequately validate the robustness of this model. For example, future results from sero-surveys that estimate the number of infections that have occurred in the third COVID-19 wave in India will allow us to better validate the accuracy of our statistical model. Additionally, data on vaccinations and on fatality rates among the vaccinated population will allow us to develop a more sophisticated understanding of COVID-19 infection incidence. This further work will be especially valuable as we enter a stage where COVID-19 remains endemic along with vaccinations becoming near-ubiquitous among the population.

Despite these limitations, our analysis provided approximate estimates of COVID-19 infection incidence in Tamil Nadu based on rather limited publicly available data. The authors hope that this study will provide some insight to academics and policymakers interested in studying the extent of COVID-19 spread in Tamil Nadu at different snapshots in time.

## 5. Conclusions

This manuscript described the analysis and modelling of COVID-19 infection incidence in the southern Indian state of Tamil Nadu. Age- and sex-disaggregated fatality data were extracted from government reports and used in conjunction with COVID-19 seroprevalence reports to derive infection fatality rates for different age groups disaggregated by sex. These IFR estimates were then further used to estimate new daily infections in Tamil Nadu for the first and second COVID waves in India. Finally, a composite statistical model combining a k-nearest neighbour regression along with a power-law curve fitting of “out-of-sample” inputs was proposed. This statistical model utilised the test positivity ratio along with daily confirmed cases to estimate the daily infection incidence. We found that the statistical model estimated the total number of infections to within 3% difference compared to the estimates from the IFR-based model.

To summarise, this manuscript proposed a statistical method that generated plausible estimates of infection incidence in Tamil Nadu and also demonstrated that these estimates were consistent with age- and sex-disaggregated IFR values that were in line with those previously reported in the literature.

## Figures and Tables

**Table 1:** Age- and Sex-wise IFR estimates for Tamil Nadu.

Age	Sex	IFR (95% CI)
18–29	M	0.003% (0.003–0.003%)
	F	0.002% (0.002–0.002%)
30–39	M	0.013% (0.013–0.013%)
	F	0.006% (0.006–0.006%)
40–49	M	0.040% (0.039–0.040%)
	F	0.017% (0.017–0.017%)
50–59	M	0.150% (0.149–0.151%)
	F	0.056% (0.055–0.056%)
60–69	M	0.363% (0.362–0.365%)
	F	0.133% (0.133–0.134%)
70+	M	0.825% (0.824–0.826%)
	F	0.258% (0.257–0.26%)

**Table 2:** IFR-based cumulative infection incidences in Wave-II.

Age	Estimate from Seroprevalence Survey	Estimate from IFR Calculations	Relative Difference (95% CI)
18–29	9,748,800	8,910,474	8.60% (8.58–8.62%)
30–39	7,718,400	11,655,338	51.01% (50.97–51.04%)
40–49	6,507,252	8,723,826	34.06% (34.03–34.1%)
50–59	4,521,874	4,827,480	6.76% (6.73–6.78%)
60–69	2,995,200	2,602,739	13.10% (13.07–13.14%)
70+	1,716,480	1,271,021	25.95% (25.89–26.02%)

## Data Availability

Publicly available datasets were analysed in this study. Tamil Nadu COVID-19 data can be found at https://stopcorona.tn.gov.in/daily-bulletin/ (accessed on 15 April 2022). Daily bulletins for Karnataka can be found at https://covid19.karnataka.gov.in/govt_bulletin/en (accessed on 15 April 2022).
